# Conservative Treatment for Isolated Superior Mesenteric Artery Dissection With Severely Narrowed True Lumen

**DOI:** 10.7759/cureus.37852

**Published:** 2023-04-19

**Authors:** Hideki Sasaki, Yukihide Numata, Shinji Kamiya, Yoshiaki Sone, Osamu Sasaki

**Affiliations:** 1 Cardiovascular Surgery, Nagoya City University East Medical Center, Nagoya, JPN; 2 Cardiology, Saitama Medical Center, Saitama Medical University, Kawagoe, JPN; 3 Internal Medicine, Kouiki Mombetsu Hospital, Mombetsu, JPN

**Keywords:** anatomy, collateral pathway, mesenteric ischemia, emergency department, isolated superior mesenteric artery dissection

## Abstract

A 59-year-old male presented to the emergency department with distressing epigastric pain after seeking medical attention at a nearby clinic three hours prior. Upon examination, the attending physician noticed edematous changes in the proximal segment of the superior mesenteric artery, and a subsequent enhanced computed tomography (CT) scan confirmed the diagnosis of an isolated dissection of the artery. Notably, the true lumen of the vessel was significantly narrowed, raising concerns for potential vascular compromise. After extensive consultation between a vascular surgeon and a radiologist, a decision was made to adopt a conservative management approach. The patient was closely monitored with meticulous bowel rest, hydration management, and carefully curated dietary modifications. Over time, subsequent CT scans revealed progressive enlargement of the true lumen, which was highly reassuring to the medical team. As a result of the expert management and diligent care provided, the patient was eventually discharged home without any adverse events or complications. This case highlights the critical role of a multidisciplinary approach in managing complex vascular pathology and underscores the importance of thoughtful clinical decision-making and meticulous monitoring in achieving favorable outcomes.

## Introduction

Isolated superior mesenteric artery dissection (ISMAD) is a rare condition, with an incidence rate of only 0.08% [1]. ISMAD refers to "intramural hematoma with intimal disruption," which occurs when there is a tear in the vessel's intima, leading to divided layers of the true and false lumen. Typical symptoms of ISMAD include abdominal and back pain along with nausea, among others. Asia appears to have a higher prevalence of ISMAD cases compared to other regions. The underlying mechanism responsible for the higher incidence of ISMAD in Asia is still a matter of ongoing research and investigation. Despite the fact that patients with this condition may seek medical attention in both emergency departments and outpatient clinics, misdiagnosis can occur, leading to a delay in treatment [2]. Fortunately, with increased awareness and utilization of computed tomography (CT), the diagnosis of ISMAD has become more frequent, and three treatment modalities, including conservative, endovascular, and surgical intervention, are chosen for individual cases [3]. In this report, we present a case of ISMAD, wherein the true lumen was severely narrowed, and the patient was managed conservatively.

## Case presentation

**Figure 1:**
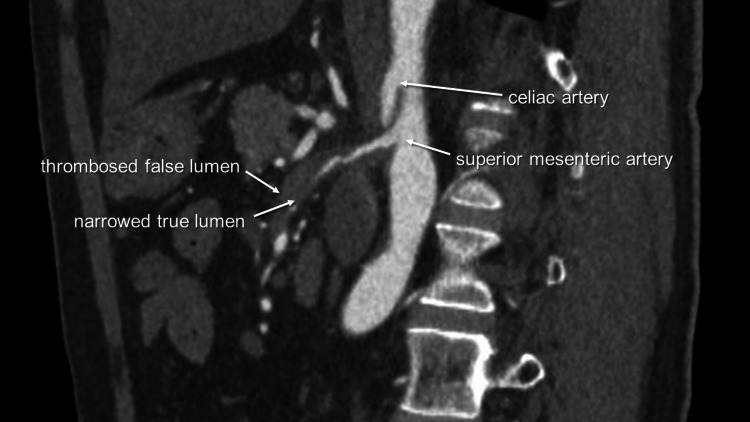
Sagittal view of the superior mesenteric artery The true lumen was severely narrowed with a thrombosed false lumen.

**Figure 2:**
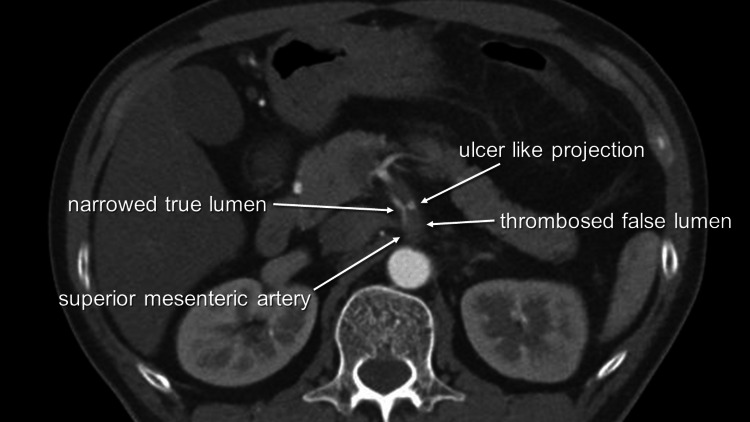
Axial view of the superior mesenteric artery An ulcer-like projection was observed 23 mm from the superior mesenteric artery origin.

A 59-year-old male patient presented with a sudden onset of abdominal pain and sought medical attention at a nearby clinic. The patient had a medical history of angina pectoris, with a stent placed in the right coronary artery six years prior and a stent placed in the left anterior descending coronary artery two weeks earlier. The patient was prescribed a regimen consisting of prasugrel and aspirin to prevent the formation of blood clots following coronary stent placement. Having no significant findings in an electrocardiogram, he was prescribed loxoprofen. Three hours later, he presented to the emergency department of our hospital with persistent pain. His blood pressure was 110/68 mmHg, heart rate was 72/minute, and body temperature was 36.3°C. Upon palpation, there was spontaneous pain but no rebound tenderness in the abdomen. Troponin levels were within normal limits, and the electrocardiogram revealed no significant findings. Abdominal X-ray showed no signs of free air, but the attending physician noticed edema around the superior mesenteric artery (SMA) in a plain CT scan. Furthermore, a contrast-enhanced CT revealed a dissection of the SMA (Figure 1). The true lumen was severely narrowed with a thrombosed false lumen, and an ulcer-like projection (ULP) was observed 23 mm from the SMA origin (Figure 2). The right colic and ileocolic arteries were visualized via collateral pathways. As a result, the patient was admitted to our hospital, and vascular surgery was consulted based on the findings. After conducting a blood test that showed a pH of 7.395 and lactate of 1.3 mmol/L, the patient's pain gradually subsided. Three hours later, lactate levels decreased to 1.1 mmol/L. The subsequent contrast-enhanced CT scan showed no change in the ULP of the proximal SMA. The true lumen was expanded, and the peripheral branches were well-enhanced. Partial wall thickening and dilatation were observed in the jejunum, indicating ischemic changes. Following a discussion between the interventional radiologist and the vascular surgeon, a decision was made to treat the patient conservatively. Consequently, we prohibited all oral intake.

**Figure 3:**
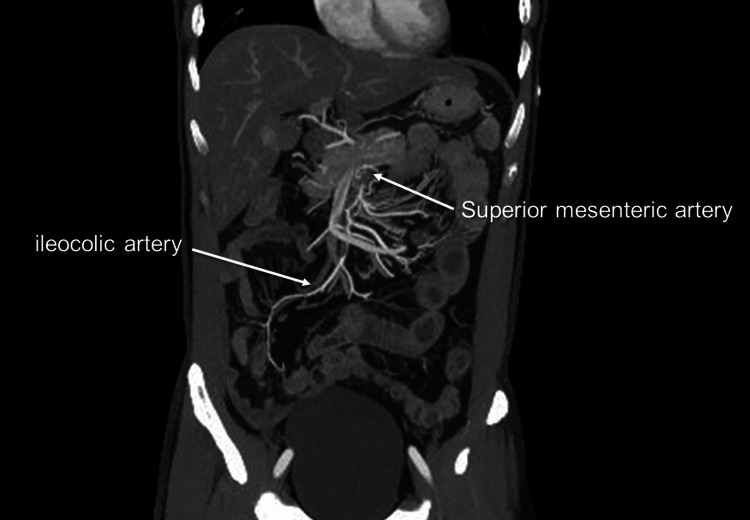
Coronal view of the superior mesenteric artery on the following day True lumen enlarged with better visualization of the ileocolic artery.

**Figure 4:**
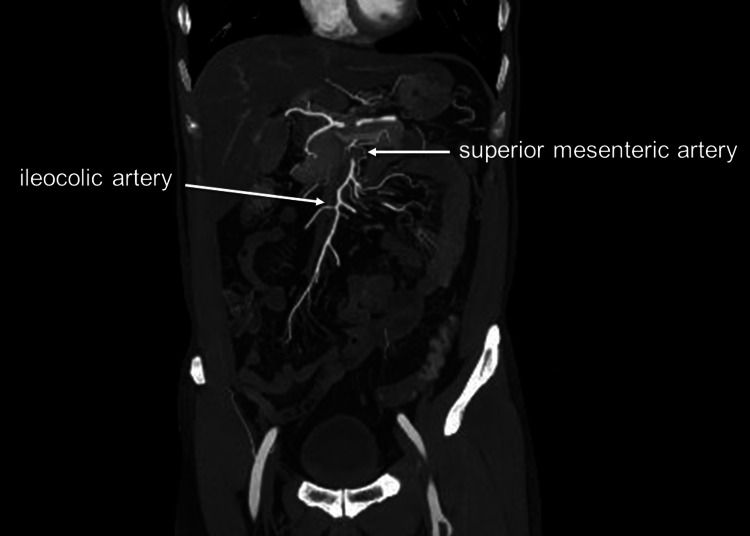
Coronal view of the superior mesenteric artery four days after the onset Better visualization of peripheral branches of the superior mesenteric artery.

On the following day, a contrast-enhanced CT scan showed no change in the ULP of the proximal SMA. Nevertheless, there was an improvement in true lumen remodeling, as well as a better contrast effect of the peripheral branches and a reduction in edematous changes in the small intestine (Figure 3). As a result, the patient was allowed to resume fluid intake and did not experience any worsening of symptoms thereafter. Two days later, a contrast-enhanced CT scan showed an enlarged true lumen with better visualization of the peripheral branches (Figure 4). The patient was started on a regular diet and did not experience any further symptoms. The patient was discharged home eight days after onset and followed up in an outpatient clinic.

## Discussion

Abdominal pain is a multifaceted symptom that often prompts patients to seek emergency medical attention or outpatient care. It can be indicative of a range of diseases, including digestive conditions such as gastroenteritis, gastrointestinal ulcers, and appendicitis, as well as vascular conditions such as abdominal aortic aneurysm, mesenteric ischemia, renal artery stenosis, and embolism. Notably, abdominal pain is not specific to ISMAD, which is characterized by various symptoms, including epigastric or umbilical discomfort and postprandial exacerbation [2]. A systematic review revealed that ISMAD, a rare condition, is predominantly observed in males, accounting for 80.6% of cases, with a mean age of 55.7 years [4]. Hypertension and smoking are often found in patients with ISMAD [2,4]. Since the first report of ISMAD in 1947, it has been recognized as a critical disease that physicians should consider as a differential diagnosis in clinical settings [5]. Its incidence has increased with the availability of contrast-enhanced CT scans. Ultrasound has also been reported to be useful for the diagnosis of ISMAD [6]. Since the classification system for ISMAD was proposed by Sakamoto, several modifications have been made [3]. The current case is classified as type III in the Sakamoto classification. Three treatment options (conservative, endovascular, and surgical) are available and applied based on individual cases. In the systematic review of 608 symptomatic patients, conservative management was administered to 438 individuals (72%), while 139 (22.8%) received the endovascular intervention, and surgical management was used for 31 cases (5%) [7]. Conversion from conservative management to either endovascular or surgical intervention was required in 12.3% and 4.4%, respectively. Although a severely narrowed true lumen was observed on the first CT scan in the current case, serial blood gas analysis confirmed no worsening of base excess and lactate levels. In addition, the second CT scan revealed remodeling of the true lumen. Therefore, we chose to take a conservative approach with careful observation of the patient. In another publication, endovascular treatment was recommended for cases where compression of the true lumen exceeded 80% [8]. However, due to the patient's gradual improvement in symptoms, we deemed a conservative approach to be appropriate in this case. Nonetheless, we remain vigilant for any further changes in the patient's condition, given the observation of partial wall thickening and intestinal dilation on the second CT scan. In cases of ISMAD, careful monitoring and management of collateral pathways are important to ensure adequate blood flow to the intestine and prevent complications. In the present scenario, it is believed that the right colic and ileocolic arteries are being visualized through these pathways. Additionally, collateral flow from the inferior mesenteric artery (IMA) may have played a pivotal role in ameliorating ischemia. As cited in a systematic review by Mann et al. [9], the marginal artery of Drummond is an important anastomotic vessel between the SMA and IMA that plays a crucial role in maintaining intestinal blood flow. It is noteworthy that mesenteric ischemia can rapidly progress to necrosis, necessitating intestinal resection in the absence of such collateral pathways. This theory can be particularly applicable to patients amenable to conservative management. In the current case, a ULP was identified at 23 mm from SMA origin, which is believed to be the entry point. At the point where SMA approached the inflection point, its sudden change in direction may have contributed to the development of an intimal tear and subsequent dissection. Research indicates that the transition of the SMA at the lower margin of the pancreas, from a fixed to a more mobile state, induces significant mechanical stress [2]. Antihypertensive medications are of utmost importance to prevent onset as well as halt aggravation in ISMAD. Controversies persist regarding the appropriate use of antiplatelet and anticoagulant drugs. While such medications may play a vital role in maintaining blood flow to the intestines and avoiding thrombosis and distal embolization, there is no definitive evidence to prove the positive effect in the treatment of ISMAD [3]. In the present case, prasugrel and aspirin have been prescribed to address the patient's coronary artery disease, and administration has commenced orally. While the prescription of these two drugs is deemed necessary, continued monitoring for the thrombosed false lumen is recommended.

In summary, the patient presenting ISMAD with a severely narrowed true lumen was treated with conservative management. Careful monitoring of the symptoms and serial enhanced CT played a pivotal role in deciding the treatment strategy for a favorable outcome.

## Conclusions

ISMAD, a life-threatening disease, may manifest as an acute abdomen in both emergency department and outpatient clinic settings. Despite its rarity, physicians should consider it as a potential differential diagnosis when encountering patients with abdominal pain. While the recommended threshold for transitioning to endovascular or bypass intervention in cases of stenosis is typically above 80%, it may be possible to continue with conservative management as long as the patient's symptoms do not deteriorate and there is no exacerbation of lactate levels.

The decision to implement treatment strategies is mainly based on the patient's symptoms, serial CT findings, and thorough discussion among surgeons and interventional radiologists.
